# Impact of comorbidities on postoperative complications in patients undergoing laparoscopy-assisted gastrectomy for gastric cancer

**DOI:** 10.1186/1471-2482-14-97

**Published:** 2014-11-22

**Authors:** Mikito Inokuchi, Keiji Kato, Hirofumi Sugita, Sho Otsuki, Kazuyuki Kojima

**Affiliations:** Department of Surgical Oncology, Tokyo Medical and Dental University, Tokyo, Japan; Department of Minimally Invasive Surgery, Tokyo Medical and Dental University, Tokyo, Japan

## Abstract

**Background:**

Comorbidity is a predictor of postoperative complications (PCs) in gastrectomy. However, it remains unclear which comorbidities are predictors of PCs in patients who undergo laparoscopy-assisted gastrectomy (LAG). Clinically, insufficient lymphadenectomy (LND) is sometimes performed in high-risk patients, although the impact on PCs and outcomes remains unclear.

**Methods:**

We retrospectively studied 529 patients with gastric cancer (GC) who underwent LAG. PCs were defined as grade 2 or higher events according to the Clavien-Dindo classification. We evaluated various comorbidities as risk factors for PCs and examined the impact of insufficient LND on PCs in patients with risky comorbidities.

**Result:**

A total of 87 (16.4%) patients had PCs. There was no PC-related death. On univariate analysis, heart disease, central nervous system (CNS) disease, liver disease, renal dysfunction, and restrictive pulmonary dysfunction were significantly associated with PCs. Both liver disease and heart disease were significant independent risk factors for PCs on multivariate analysis (odds ratio [OR] = 3.25, p = 0.022; OR = 2.36, p = 0.017, respectively). In patients with one or more risky comorbidity, insufficient LND did not significantly decrease PCs (p = 0.42) or shorten GC-specific survival (p = 0.25).

**Conclusion:**

In patients who undergo LAG for GC, the presence of heart disease or liver disease is an independent risk factor for PC. Insufficient LND (for example, D1+ for advanced GC) might be permissible in high-risk patients, because although it did not reduce PCs, it had no negative impact on GC-specific survival.

## Background

Gastric cancer (GC) is the fourth most common malignancy [1]. At present, the worldwide treatment of choice for GC is complete surgical removal of the tumor and adjacent lymph nodes. Surgical outcomes are influenced by various factors, including patients’ characteristics and concurrent disease, type of operation, and quality of care. Postoperative complications (PCs) negatively affect the quality of life of patients who undergo gastrectomy and can even be life-threatening. Identification of risk factors for PCs might help to reduce such complications, and many studies have attempted to evaluate risk factors for PCs associated with various procedures. Comorbidity has been reported to be a predictor of PCs in patients who receive gastrectomy for GC [2–5]. However, what types of comorbidities are associated with the highest risk of PCs in patients who undergo gastrectomy remains to be fully defined. Risk factors probably differ between abdominal (surgical) and non-abdominal (medical) PCs. The primary objective of study was to clarify comorbidities associated with PCs in laparoscopy-assisted gastrectomy (LAG), a procedure for less invasive surgery increasingly used throughout the world. Clarifying specific comorbidities might contribute to improved treatment strategies for GC.

Scoring systems such as the Estimation of Physiologic Ability and Surgical Stress (E-PASS) score and the Physiologic and Operative Severity Score for the enUmeration of Mortality and morbidity (POSSUM) are useful for predicting the risks of mortality and morbidity after various operations [6, 7], although they are not commonly used in clinical practice. In patients with comorbidities likely to adversely affect postoperative outcomes, standardized treatments, such as gastrectomy with D2 lymphadenectomy (LND) for advanced GC, tend to be avoided by surgeons. However, criteria for the selection of patients who should undergo insufficient LND and the impacts of insufficient LND on PCs and survival in high-risk patients remain to be defined. The secondary objective of this study was to evaluate the outcomes of high-risk patients who underwent insufficient LND. We verified whether insufficient LND negatively affects postoperative survival in this retrospective study.

## Methods

We retrospectively identified 529 consecutive patients who underwent LAG with LND for pathological stage I to III GC in our hospital between 2003 and 2012. Patients who underwent thoracolaparotomy, emergency surgery, incomplete tumor resection, and combined operations for other malignancies were excluded. The present study was in compliance with the Declaration of Helsinki, and was approved by the ethics committee of Tokyo Medical and Dental University. In principle, early-stage GC was treated by LAG in accordance with the treatment guidelines of the Japanese Gastric Cancer Association [8]. The extent of LND was retrospectively classified as D1, D1+ (α or β), or D2 in accordance with the treatment guidelines, version 2 [8]. However, reduced LND was performed in patients with severe comorbidities. In patients who underwent LAG, carbon dioxide pneumoperitoneum was maintained at 10 mm Hg, and a 4- to 5-cm incision was made in the upper abdomen or navel to remove tissue specimens and conduct anastomosis. For lymph node dissection, we used harmonic scissors and monopolar and bipolar electric cautery devices. All patients received systemic antibiotics (a first-generation cephem) several times on the day of surgery. The nasogastric tube was left in place until postoperative day 1 according to our protocol.

All patients preoperatively underwent venous blood analysis (including hemoglobin, serum albumin, and creatinine), electrocardiography, chest radiography, and pulmonary function testing, including vital capacity (VC), forced expiratory volume in 1 second (FEV1), and forced vital capacity (FVC). The results of these examinations were retrieved from the patients’ electronic medical records. The following variables were obtained from our prospective GC database: patient age and gender; body mass index (BMI); comorbidities; regular use of steroids; tumor characteristics; extent of lymph-node dissection; operation time; estimated blood loss; and PCs. All comorbidities other than pulmonary and renal dysfunction were defined as conditions that required treatment. For example, heart disease included ischemic disease treated by interventional procedures, atrial fibrillation requiring anticoagulant treatment, and congenital cardiac failure treated by medication. Liver disease included both cirrhosis and chronic hepatitis treated by medication. Pulmonary dysfunction was classified into two categories on basis of the results of preoperative spirometry. Restrictive pulmonary dysfunction was defined as a predicted VC of less than 80%, and chronic obstructive pulmonary disease (COPD) was defined as an FEV1/FVC ratio of less than 0.70. Renal dysfunction was defined as a serum creatinine concentration higher than the upper limit of normal according to our hospital’s criteria (>1.1 mg/dL in males and >0.8 mg/dL in females). Anemia was defined according to the World Health Organization (WHO) criteria (<13 g/dL in males and <12 g/dL in females). Hypoalbuminemia was defined as a serum albumin concentration of less than 3.5 g/dL. In addition, some comorbidities were classified into two groups according to severity.

All patients were followed up until June 2013. The median follow-up was 52 months (5.5-126). A total of 59 (11.1%) patients died, 19 (3.6%) had recurrence of GC, and 40 (7.6%) died of other causes. Thirty-two patients (6.0%) died of benign diseases, such as cardiac, pulmonary, hepatic, and renal disease.

Patients’ characteristics and surgical outcomes are shown in Table 1. In this study, PCs were defined as grade 2 or higher events according to the Clavien-Dindo classification that occurred within 30 days after gastrectomy [9]. In addition, PCs were classified into either abdominal or non-abdominal complications.

**Table 1:** **Patients’ characteristics and surgical outcomes**

	n%
Gender	
Male	380 (71.8)
Female	149 (28.2)
Age mean ± SD	64.9 ± 11.5
Body mass index (kg/m^2^) mean ± SD	22.9 ± 3.1
Comorbidities	326 (61.6)
Heart disease	50 (9.5)
Ischemic disease	24 (4.5)
Arrhythmia	24 (4.5)
Congenital cardiac failure	3 (0.6)
Others	7 (1.3)
CNS disease	39 (7.4)
Cerebrovascular disease	30 (5.7)
Neurodegenerative disease	6 (1.1)
Others	3 (0.6)
Liver disease	21 (4.0)
Liver cirrhosis	8 (1.5)
Chronic hepatitis	13 (2.4)
Renal dysfunction^a)^	54 (10.2)
Pulmonary dysfunction	124 (23.4)
Restrictive pulmonary dysfunction^b)^	25 (4.7)
COPD	112 (21.2)
Diabetes mellitus	67 (12.7)
Hypertension	184 (34.8)
Other disease	45 (8.5)
Anemia^c)^	131 (24.8)
Hyoalbuminemia^d)^	8 (1.5)
Type of gastrectomy	
Total	78 (14.7)
Proximal	34 (6.4)
Distal	417 (78.8)
Extent of LND	
D1	4 (0.8)
D1+	448 (84.7)
D2	77 (14.6)
Combined resection	54 (10.2)
Gallbladder	39 (7.4)
Spleen	13 (2.5)
Intestine or colon	2 (0.4)
Operating time (min) mean ± SD	287 ± 75
Bleeding (g) median (range)	72 (0 – 2492)
Pathological tumor stage	
I	438 (82.8)
II	60 (11.3)
III	31 (5.9)

SD standard deviation, CNS central nervous system.

COPD: chronic obstructive pulmonary disease, LND: lymph node dissection

^a)^serum creatinine concentration higher than the upper limit of normal at our hospital, >1.10 in males and >0.80 in females.

^b)^predicted vital capacity <80%.

^c)^decreased hemoglobin, <13 g/dL in males and <12 g/dL in females.

^d)^decreased serum albumin <3.5 g/dL.

Next, we identified patients who had comorbidities that were risk factor for PCs. They were divided into two groups: patients underwent insufficient LND and those underwent sufficient LND. Insufficient LND included both insufficient D1+ dissection in pathological stage IA cancer with submucosal invasion and insufficient D2 dissection in pathological stage IB or more advanced cancer. We compared clinical outcomes between the patients who underwent insufficient LND and those underwent sufficient LND.

### Statistical analysis

All variables were classified into two categories and were compared with the use of the chi-square test or Fisher’s exact test, as appropriate. Multivariate analysis was carried out by binary logistic multiple regression testing using dummy variables. Seven patients (1.3%) were excluded from the multivariate analysis because of missing data. Survival was measured from the date of performing LAG to the latest follow-up date or the date of death. Kaplan-Meier curves were plotted to assess the effect of insufficient LND for patients with any risky comorbidity on survival. Different curves of survival were compared using the log-rank test. P values of <0.05 were considered to indicate statistical significance. All analyses were performed with the statistical software package SPSS 20 (SPSS Japan Inc., Tokyo, Japan).

## Results

A total of 87 (16.4%) patients had PCs. There was no PC-related death. Overall, 66 (12.5%) patients had abdominal complications, 5 (0.9%) had cardiac complications, 10 (1.9%) had pulmonary complications, and 15 (2.8%) had other complications. The details of the PCs are shown in Table 2. As for surgical factors, D2 LND, D1+ LND, and D1 LND were performed in 77 (14.6%), 448 (84.7%), and 4 (0.8%) patients, respectively. Total gastrectomy, proximal gastrectomy, and distal gastrectomy were performed in 78 (14.7%), 34 (6.4%), and 417 (78.8%) patients.

**Table 2:** **Postoperative complications**

	n %	Grade 2/3/4/5
Total	87 (16.4)	48/34/5/0
Abdominal	66 (12.5)	31/33/2/0
Anastomotic leakage	8 (1.5)	0/7/1/0
Pancreatic fistula	5 (0.9)	1/4/0/0
Abdominal abscess	14 (2.6)	4/10/0/0
Anastomotic stenosis	14 (2.6)	3/11/0/0
Ileus	9 (1.7)	5/3/1/0
Gastric stasis	5 (0.9)	5/0/0/0
Postoperative bleeding	4 (0.8)	3/1/0/0
Ascites	4 (0.8)	3/1/0/0
Cholecystitis	2 (0.4)	1/1/0/0
Cholerrhagia	1 (0.2)	0/1/0/0
Reflux esophagitis	2 (0.4)	2/0/0/0
Enteritis	1 (0.2)	1/0/0/0
Wound infection	6 (1.1)	6/0/0/0
Non-abdominal	30 (0.9)	25/2/3/0
Ischemic attack	1 (0.2)	0/0/1/0
Arrhythmia	4 (0.8)	4/0/0/0
Pneumonia	6 (1.1)	5/1/0/0
ARDS	2 (0.4)	0/0/2/0
Atelectasis	2 (0.4)	2/0/0/0
Urinary tract infection	2 (0.4)	2/0/0/0
Infection of venous catheter	1 (0.2)	1/0/0/0
Deep vein thrombosis	1 (0.2)	0/1/0/0
Cerebral bleeding	1 (0.2)	0/0/1/0
Delirium	12 (2.3)	12/0/0/0

ARDS acute respiratory distress syndrome.

### All PCs

On univariate analysis, PCs were significantly associated with many factors: male gender, higher age (≥75 years), heart disease, CNS disease, liver disease, renal dysfunction, restrictive pulmonary dysfunction, anemia, regular use of steroids, total gastrectomy, combined resection of other organ (except gallbladder), extended operating time (≥300 minutes), and higher operative bleeding volume (≥300 g) (Table 3). Only 5 patients (0.9%) received transfusion, and transfusion was not assessed in this study. Next, we evaluated independent risk factors for PCs using a multivariate model adjusted for all of the above risk factors (Table 4). Finally, both liver disease and heart disease were independent risk factors significantly related to PCs on multivariate analysis (odds ratio [OR] = 3.25, 95% confidential interval [CI]: 1.18-8.91, p = 0.022; OR = 2.36, 95% CI: 1.17-4.76, p = 0.017, respectively). The following factors showed a trend toward being risk factors on multivariate analysis: CNS disease (OR = 2.24, 95% CI: 1.00-5.01, p = 0.050), renal dysfunction (OR = 2.01, 95% CI: 0.98-4.13, p = 0.058), male gender (OR = 1.75, 95% CI: 0.93-3.29, p = 0.082), higher age (OR = 1.70, 95% CI: 0.95-3.03, p = 0.075), combined resection (OR = 2.85, 95% CI: 0.88-9.27, p = 0.081), and extended operating time (OR = 1.61, 95% CI: 0.95-2.73, p = 0.079).

**Table 3:** **Univariate analysis for risk factors of PCs in LAG**

		All			Abdominal		Non-abdominal	
		n	n (%)	p	n (%)	p	n (%)	p
Gender	male	380	71 (18.7)	0.027	53 (13.9)	0.10	27 (7.1)	0.023
	female	149	16 (10.7)		13 (10.8)		3 (2.0)	
Age	≥75	117	34(29.0)	<0.001	27 (23.1)	<0.001	12 (11.4)	0.015
	<75	412	53 (12.9)		39 (9.5)		18 (4.4)	
Body mass index	≥25 (kg/m^2^)	136	18 (13.2)	0.24	15 (11.0)	0.55	6 (4.4)	0.46
	<25	393	69 (17.6)		51 (13.0)		24 (6.1)	
Heart disease	yes	50	18 (36.0)	<0.001	13 (26.0)	0.002	10 (20.0)	<0.001
	no	479	69 (14.4)		53 (11.1)		20 (4.1)	
CNS disease	yes	39	13 (33.3)	0.003	10 (25.6)	0.020	7 (17.9)	0.004
	no	490	74 (15.1)		56 (11.4)		23 (4.7)	
Liver disease	yes	21	8 (38.1)	0.013	8 (38.1	<0.001	2 (9.5)	0.34
	no	508	79 (15.6)		58 (11.4)		28 (5.5)	
Diabetes mellitus	yes	67	12 (17.9)	0.73	10 (14.9)	0.52	5 (7.5)	0.57
	no	462	75 (16.2)		56 (12.1)		25 (5.4)	
Hypertension	yes	184	34 (18.5)	0.36	25 (13.6	0.57	13 (7.1)	0.31
	no	345	53 (15.4)		41 (11.9)		17 (4.9)	
Renal dysfunction	yes	54	17 (31.5)	0.002	14 (25.9)	0.002	6 (11.1)	0.11
	no	475	70 (14.7)		52 (12.3)		24 (5.1)	
Restrictive pulmonary dysfunction	yes	25	10 (40.0)	0.003	7 (28.0)	0.026	6 (24.0)	0.001
	no	498	75 (15.0)		58 (13.2)		23 (4.6)	
	not evaluated	6	2		1		1	
COPD	yes	112	17 (15.2)	0.73	11 (9.8)	0.35	8 (7.1)	0.41
	no	411	68 (16.5)		54 (13.1)		21 (5.1)	
	not evaluated	6	2		1		1	
Anemia	yes	131	29 (22.1)	0.043	22 (16.8)	0.085	12 (9.2)	0.047
	no	398	58 (14.6)		44 (11.1)		18 (4.5)	
Hypoalbuminemia	yes	8	3 (37.5)	0.13	2 (25.0)	0.26	2 (25.0)	0.071
	no	519	84 (16.2)		64 (12.3)		28 (5.4)	
	not evaluated	2	0		0		0	
Regular use of steroid	yes	9	4 (44.4)	0.045	2 (22.2)	0.31	2 (22.2)	0.087
	no	520	83 (16.0)		64 (12.3)		28 (5.4)	
Type of resection	total or proximal	112	29 (25.9)	0.002	20 (17.9)	0.052	13 (11.6)	0.002
	distal	417	58 (13.9)		46 (12.4)		17(4.1)	
Extent of lymph node dissection	D2	77	15 (19.5)	0.44	10 (13.0)	0.88	5 (6.5)	0.79
	D1+ or D1	452	72 (15.9)		56 (12.4)		25 (5.5)	
Combined resection	yes	16	6 (37.5)	0.033	4 (25.0)	0.13	2 (12.5)	0.23
	no or gallbladder	513	81 (15.8)		62 (12.1)		28 (5.5)	
Operating time	≥300 (min)	253	52 (20.6)	0.015	39 (15.4)	0.050	19 (7.5)	0.080
	<300	276	35 (12.7)		27 (9.8)		11 (4.0)	
Estimated bleeding	≥500(g)	43	12 (27.9)	0.031	10 (23.3)	0.026	4 (9.3)	0.28
	<500	485	74 (15.2)		56 (11.5)		25 (5.2)	
	unknown	1	1		0		1

**Table 4:** **Multivariate analysis of risk factors for PCs in LAG**

	All PCs			Abdominal PCs			Non-abdominal PCs		
	OR	95% CI	p	OR	95% CI	p	OR	95% CI	p
Male gender	1.75	0.93-3.29	0.082	1.70	0.91-3.17	0.099	1.57	0.86-2.89	0.15
Higher age (≥75)	1.70	0.95-3.03	0.075	1.66	0.93-2.96	0.086	1.84	1.04-3.26	0.036
Heart disease	2.36	1.17-4.76	0.017	2.40	1.20-4.82	0.013	2.31	1.15-4.64	0.019
CNS disease	2.24	1.00-5.01	0.050	2.11	0.94-4.73	0.070	1.99	0.88-4.49	0.097
Liver disease	3.25	1.18-8.91	0.022	3.10	1.13-8.47	0.028			
Renal dysfunction	2.01	0.98-4.13	0.058	2.13	1.06-4.29	0.035			
Restrictive pulmonary dysfunction	2.08	0.81-5.34	0.13	1.95	0.76-4.99	0.16	2.12	0.83-5.42	0.12
Anemia	0.93	0.51-1.69	0.81	1.04	0.58-1.85	0.90	1.02	0.57-1.83	0.95
Hypoalbuminemia							1.53	0.21-11.2	0.67
Regular use of steroids	2.93	0.58-14.8	0.19				4.47	1.04-19.3	0.045
Total or proximal gastrectomy	1.39	0.76-2.55	0.29	1.52	0.85-2.73	0.16	1.73	0.99-3.00	0.052
Combined resection	2.85	0.88-9.27	0.081						
Extended operating time (≥300 min)	1.61	0.95-2.73	0.079	1.57	0.93-2.64	0.093	1.71	1.02-2.86	0.043
Higher operative bleeding (≥500 g)	1.10	0.48-2.52	0.83	1.22	0.54-2.76	0.64			

PCs postoperative complications, LAG laparoscopy-assisted gastrectomy.

### Subcategorized PCs

For analysis, PCs were subcategorized into abdominal and non-abdominal (cardiac, pulmonary, etc.) PCs. Abdominal PCs occurred in 75.9% of the patients with PCs. Abdominal PCs were significantly associated with many factors on univariate analysis (Table 3). Multivariate analysis showed 3 independent predictors of abdominal PCs: liver disease (OR = 3.10, 95% CI: 1.13-8.47, p = 0.028), heart disease (OR = 2.40, 95% CI: 1.20-4.82, p = 0.013), and renal dysfunction (OR = 2.13, 95% CI: 1.06-4.29, p = 0.035). Extended operating time and higher operative bleeding were not significant predictors on multivariate analysis (OR = 1.57, 95% CI: 0.93-2.64, p = 0.093; OR = 1.22, 95% CI: 0.54-2.76, p = 0.64, respectively) (Table 4).

Non-abdominal PCs were also significantly associated with many factors on univariate analysis (Table 3). Heart disease was also an independent risk factor for non-abdominal PCs (OR = 2.31, 95% CI: 1.15-4.64, p = 0.019) on multivariate analysis. Three other factors were independent predictors of non-abdominal PCs: higher age (OR = 1.84, 95% CI: 1.04-3.26, p = 0.036), regular use of steroids (OR = 4.47, 95% CI: 1.04-19.3, p = 0.045), and extended operating time (OR = 1.71, 95% CI: 1.02-2.86, p = 0.043) (Table 4).

### Relation between PCs and severity of comorbidities

The severity of each comorbidity was not significantly related to an increased incidence of PCs, although a high rate of PCs was found in patients with liver cirrhosis (Table 5).

**Table 5:** **Relationship between severity of each comorbidity and PCs**

Comorbidity	Classification by severity	PCs		
		n	n (%)	p
Heart disease	surgical or interventional	19	5 (26)	0.26
	only medication	31	13 (42)	
COPD	stage 3 or 4*	8	0 (0)	0.60
	stage 1 or 2	104	17 (16)	
CNS disease	paralysis	8	2 (25)	0.69
	no paralysis	31	11 (35)	
Liver disease	cirrhosis	8	5 (63)	0.16
	hepatitis	13	3 (23)	
Renal dysfunction	dialysis	5	2 (40)	0.65
	no dialysis	49	15 (31)	
Diabetes mellitus	regular use of insulin	16	4 (25)	0.46
	oral medication	51	8 (16)	

*The stage of COPD is defined by Global Initiative for Chronic Obstructive Lung Disease.

### Impact of insufficient LND on PCs and survival of patients with any risky comorbidity

We assessed the impact of insufficient LND (as defined in the Methods section) on PCs and survival in patients with the following risky comorbidities: heart disease, CNS disease, liver disease, renal dysfunction, and restrictive pulmonary dysfunction, all of which were significantly associated with PCs. A total of 149 patients (28% of all patients) had these risky comorbidities, and 42 (28%) of these patients underwent insufficient LND. The characteristics of the patients included in this portion of the study are shown in Table 6. The patients who underwent insufficient LND had a more advanced stage of GC (p < 0.001). The incidences of all PCs and of abdominal PCs were similar in the patients who underwent insufficient LND and those who underwent sufficient LND (29% vs 30%, p = 0.87; 19% vs 25%, p = 0.42, respectively). However, the incidence of non-abdominal PCs was significantly higher in the patients who underwent insufficient LND than in those who underwent sufficient LND (21% vs 8%, p = 0.028) (Table 6). The overall survival rate was slightly, but not significantly lower in patients who received insufficient LND (60.6% vs 79.0%, p = 0.24). However, GC-specific survival was similar in the two groups (90.6% vs 94.4%, p = 0.25), regardless of the fact that patients who underwent insufficient LND had a significantly more advanced stage of GC than those who underwent sufficient LND (Table 7).

**Table 6:** **Comparison between insufficient LND and sufficient LND in patients with any risky comorbidity**

		Insufficient LND	Sufficient LND	
		n = 42	n = 107	
		n (%)	n (%)	p
Age	≥75	18 (43)	45 (42)	0.93
	<75	24 (57)	62 (58)	
Gender	male	34 (81)	76 (71)	0.22
	female	8 (19)	34 (29)	
Tumor stage	I	22 (52)	96 (90)	<0.001
	II	14 (33)	7 (7)	
	III	6 (14)	4 (4)	
LND	D1	2 (5)	1 (0.9)	0.015
	D1+	40 (95)	91 (85)	
	D2	0 (0)	15 (14)	
No. of risky comorbidity	1	32 (76)	83 (78)	0.73
	2	9 (21)	19 (18)	
	≥3	1 (2)	5 (5)	
All PCs		12 (29)	32 (30)	0.87
Abdominal PCs		8 (19)	27 (25)	0.42
Non-abdominal PCs		9 (21)	9 (8)	0.028

**Table 7:** **OS and DSS in patients with any risky comorbidity**

		5-year OS (%)	p	5-year DSS (%)	p
Age	<75	77.3	0.069	92.6	0.94
	≥75	44.9		94.4	
Gender	male	76.5	0.43	100.0	0.22
	female	68.5		80.8	
Tumor stage	I	81.8	<0.001^a^	100.0	<0.001^a,b^
	II	64.6	0.006^c^	80.8	0.022^c^
	III	25.0		42.2	
No. of risky comorbidity	1	73.3	0.19	92.8	0.61
	≥2	45.1		95.0	
LND	sufficient	79.0	0.24	94.4	0.25
	insufficient	60.6		90.6	

OS; overall survival, DSS; disease-specific survival.

^a^stage I vs III, ^b^stage I vs II, ^c^stage II vs III.

## Discussion

Our results showed that heart, CNS, liver, renal, and pulmonary comorbidities or dysfunctions were risk factors for PCs after radical gastrectomy. Heart disease and liver disease were independent risk factors for PCs in the present study, consistent with the results of a previous study of gastrectomy with D2 LND by Jeong et al. [2]. Heart disease and liver disease might be common risk factors after gastrectomy. However, Jeong et al. did not mention renal or pulmonary dysfunction, and the rates of comorbidities such as heart disease (4.6%) and neurological disease (2.2%) were lower than those in our study. Moreover, the rate of laparoscopic surgery was only 9.0% in their study. Another study found that liver cirrhosis and hypertension were independent risk factors for PCs in patients ≥70 years of age who underwent gastrectomy [5]. Most patients underwent D1+ LND and distal gastrectomy in our study, because the Japanese guidelines recommend LAG for the treatment of early GC. Therefore, our results would most likely differ somewhat from those of similar studies performed in Western countries owing to differences in the most common sites of GC and the disease stage at diagnosis as compared with Japan.

In previous studies of only LAG in patients with mainly early gastric cancer, higher age (≥60 years), male gender of the patient, and type of resection or reconstruction procedure were predictors of local PCs, and inadequate experience of the operator was a predictor of systemic PCs [3, 4]. Higher age was not a significant predictor of PCs in other studies of LAG [10–13], while higher age was significantly associated with non-abdominal PCs in this study. In the present study, 4 surgeons qualified in LAG performed all LAG procedures. The experience of the surgeons thus did not affect clinical outcomes. Our study had several limitations. Most important, it was a single-center study performed by experts in LAG. Our results thus might not be applicable to general hospitals. A pooled analysis or a multicenter study involving surgeons with various degrees of experience is necessary to identify common risk factors for gastrectomy.

In three studies of D2 LND including many patients who underwent OG, multiple-organ resection, advanced disease stage, extended operating time (≥180 or 200 minutes), higher age (≥50 years), male gender, higher BMI (≥25), and type of reconstruction were significant independent predictors of PCs [2, 14, 15]. In a randomized clinical trial of OG with D2 or more extended LND, higher age (>65 years), pancreatectomy, and extended operating time (>297 minutes) were independent risk factors for PCs [16]. In another study of open gastrectomy with various extents of lymph-node dissection, splenectomy or an extended operative time (≥360 minutes) was a risk factor for abdominal PCs [17].

Obesity is an established operative risk factor, but patients with a BMI of ≥30, defined as obese by the WHO, are uncommon in Asia. Obesity has therefore been an uncertain predictor of PCs in patients who undergo LAG [18–21]. Diabetes mellitus is a known risk factor for PCs after pancreaticoduodenectomy and hepatectomy [22, 23], while Jeong et al. found no relation between diabetes mellitus and PCs after gastrectomy [5]. Preoperative strict diabetic control by diabetologists for about 2 weeks in patients with severe diabetes mellitus in our hospital might have resulted in the favorable postoperative course. COPD is a risk factor for postoperative pulmonary complications after non-thoracic surgery [24]. COPD was not associated with postoperative pulmonary complications in our study or in a previous study including patients who received open gastrectomy [25]. Preoperative smoking cessation for about 3 to 4 weeks in all patients and breathing exercises in patients with severe COPD might have contributed to the low incidence of pulmonary complications (10 patients, 1.8%), and 8 (1.5%) patients with 3 or more severe COPDs had no pulmonary complications in this study.

Nomograms established from preoperative data can facilitate the design of treatment strategies, but require a large volume of data from multiple centers. The Charlson comorbidity index (CCI) was developed to predict 10-year mortality for patients with a range of comorbidities [26]. Park et al. showed that the age-adjusted CCI was a useful predictor of systemic complications after LAG [27]. E-PASS and POSSUM predict the risks of mortality and morbidity after various operations, and the latter has been employed in patients undergoing gastrectomy [6, 7]. However, these systems have not been routinely used in clinical practice, and many surgeons base treatment strategies on the severity of comorbidities or age of the patient. Clinically, reduced insufficient LND is often performed in patients with severe comorbidity or higher age, although criteria defining the need for more conservative procedures remain unclear. The indications for insufficient LND in risky patients were decided by consensus among a team of gastrointestinal surgeons in our hospital and were primarily based on the general condition of risky patients; we had no predefined criteria for such indications. We performed at least D1+ LND in risky patients who had a preoperative diagnosis of advanced GC. Insufficient LND did not reduce PCs in patients with risky comorbidities. In contrast, cardiac or pulmonary PCs increased in this study. However, if all patients had undergone sufficient LND, more PCs might have occurred. In addition, insufficient LND did not significantly shorten GC-specific survival in patients with any risky comorbidity. Insufficient LND, such as D1+ LND for advanced cancer, may thus be permissible in high-risk patients. A prospective randomized controlled trial would be the most reliable means of objectively evaluating the advantages and disadvantages of insufficient LND, but would be risky to perform in patients with severe comorbidities. A multicenter study or a pooled analysis is considered a better means of resolving this issue in the future. In the present study, the severity of comorbidities was not significantly related to the incidence of PCs. This finding might be attributed to the fact that few patients with severe comorbidities were allowed to receive prolonged general anesthesia. In such patients, we performed local resection with limited sampling of lymph nodes, endoscopic resection without LND, or sometimes withheld anticancer treatments.

## Conclusions

Heart, CNS, liver, renal, and pulmonary comorbidities or dysfunctions were risk factors for PCs after LAG in patients with GC. Heart disease and liver disease were independent risk factors for PCs. In high-risk patients, insufficient LND did not decrease PCs, but had no negative impact on GC-specific survival. Insufficient LND, such as D1+ LND for advanced GC, might thus be permissible in this subgroup of patients.

### Consent

Written informed consent was obtained from the patients or their guardian/parent/next of kin for this postoperative research.
